# Supplementary material for: The causal role of circulating inflammatory markers in osteoporosis: a bidirectional Mendelian randomized study
Source: Front Immunol. 2024 Jul 18;15:1412298. doi: 10.3389/fimmu.2024.1412298 (PMC11291241; doi:10.3389/fimmu.2024.1412298)
Supplement: Supplementary file 1 [file DataSheet_1.zip › supplementary material/OSTEOPOROSIS-CIM/GCST90274812.tsv.gz_forest.pdf]

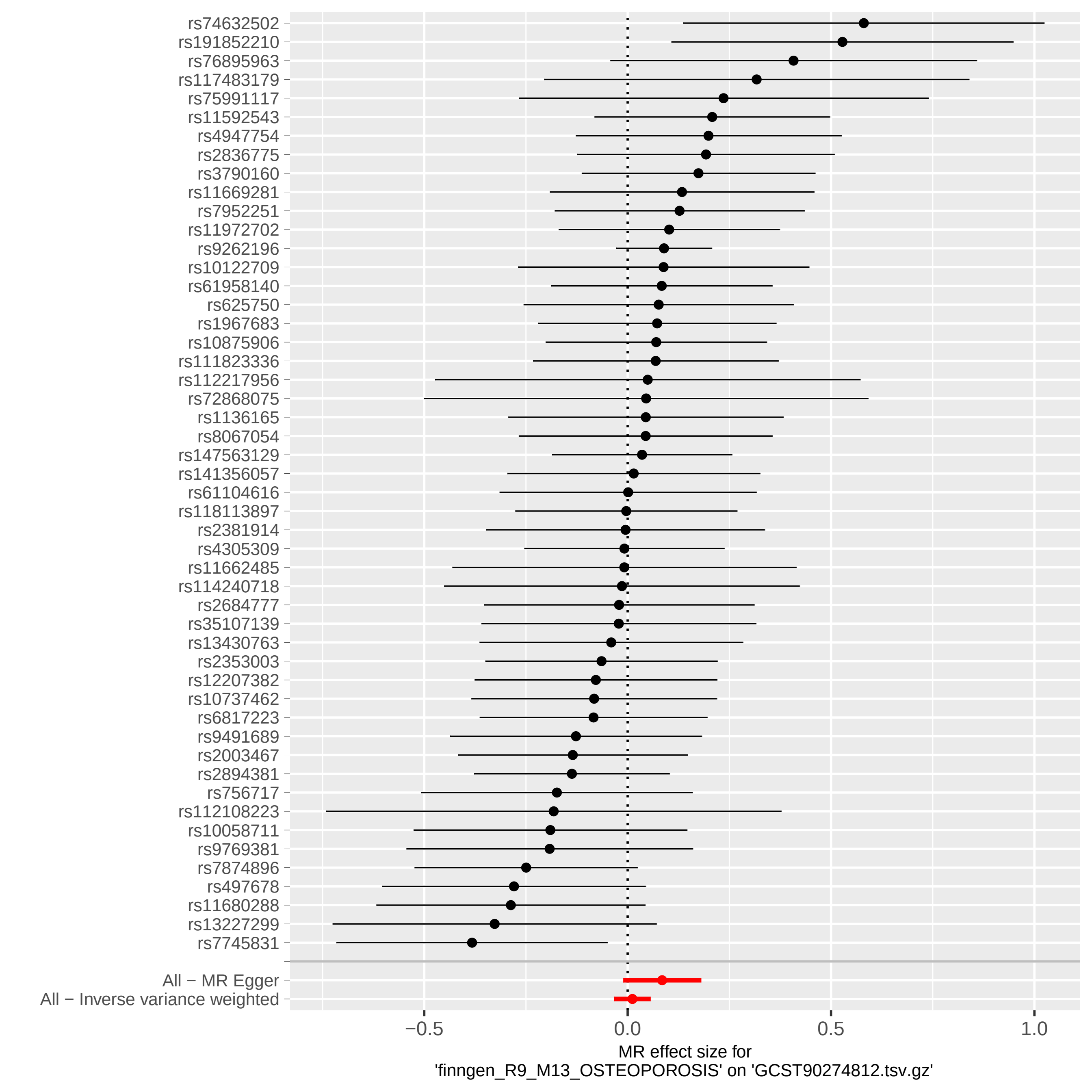

rs74632502
rs191852210
rs76895963
rs117483179
rs75991117
rs11592543
rs4947754
rs2836775
rs3790160
rs11669281
rs7952251
rs11972702
rs9262196
rs10122709
rs61958140
rs625750
rs1967683
rs10875906
rs111823336
rs112217956
rs72868075
rs1136165
rs8067054
rs147563129
rs141356057
rs61104616
rs118113897
rs2381914
rs4305309
rs11662485
rs114240718
rs2684777
rs35107139
rs13430763
rs2353003
rs12207382
rs10737462
rs6817223
rs9491689
rs2003467
rs2894381
rs756717
rs112108223
rs10058711
rs9769381
rs7874896
rs497678
rs11680288
rs13227299
rs7745831
All − MR Egger
All − Inverse variance weighted
−0.5
0.0
0.5
1.0
MR effect size for
'finngen_R9_M13_OSTEOPOROSIS' on 'GCST90274812.tsv.gz'
